# State-Level Variability in Location of Death of Patients with End-Stage Liver Disease

**DOI:** 10.1007/s10620-025-09433-w

**Published:** 2025-10-08

**Authors:** Julia Meguro, Michael Huber, David Goldberg

**Affiliations:** 1 Department of Medicine, University of Illinois, Chicago, USA; 2 Division of Geriatrics and Palliative Medicine, Department of Medicine, University of Miami Miller School of Medicine, Miami, USA; 3 Division of Digestive Health and Liver Diseases, Department of Medicine, University of Miami Miller School of Medicine, Don Soffer Clinical Research Center, 1120 NW 14th St., Room 807, Miami, FL 33136 USA

**Keywords:** End-stage liver disease, Hepatocellular carcinoma, Hospice, End-of-life care

## Abstract

**Purpose:**

Although deaths from end-stage liver disease (ESLD) and hepatocellular carcinoma (HCC) in the United States increasingly occur at home or in hospice, inpatient medical facility deaths remain high. Despite the decrease in in-hospital deaths for all causes, non-White decedents are more likely than White decedents to die in a hospital setting. This study aimed to determine state-level variability in the location of death among patients with ESLD and HCC and to assess racial/ethnic differences in these patterns, focusing on Black, White, and Hispanic/Latino patients.

**Methods:**

A retrospective cross-sectional analysis was conducted using 2018–2022 data from the Centers for Disease Control and Prevention Wide-Ranging OnLine Data for Epidemiologic Research. The proportion of patients with ESLD, HCC, and both conditions who died at an inpatient medical facility, home, hospice facilities, and a combination of both home and hospice was calculated, stratified by race/ethnicity. Mapping was utilized to compare these proportions across the US.

**Results:**

There was notable geographic variation in the location of death across all groups. Black and Hispanic/Latino patients with ESLD and HCC more frequently died in inpatient facilities compared to White patients. A statistically significant positive correlation was observed between the number of registered hospice agencies in a state and the proportion of deaths occurring at home among White (Spearman’s *ρ* = 0.33, *p* = 0.02) and Hispanic/Latino patients (Spearman’s *ρ* = 0.38, *p* = 0.01).

**Conclusions:**

Future research should investigate factors driving interstate variability and racial differences in end-of-life care for ESLD and HCC patients, which may include hospice availability and the presence of palliative care laws. Strategies to reduce these differences and enhance access to quality end-of-life care for all, particularly for racial/ethnic minorities, are needed.

**Supplementary Information:**

The online version contains supplementary material available at 10.1007/s10620-025-09433-w.

## Introduction

Hospice is widely regarded as the optimal model of care for individuals with life-limiting illnesses, serving as an indicator of high-quality end-of-life care (EOL) and aligning with the goal of providing appropriate, patient-centered care [1]. There is increased interest in the utilization of hospice and palliative care for patients with end-stage liver disease (ESLD), who experience physical, psychological, and social impairments [2]. Palliation of symptoms at home is preferred over invasive therapies and hospitalization at the end of life by most older adults living with chronic illnesses in the US, and hospice care for patients with ESLD can decrease healthcare utilization and costs [2,3].

From 2003 to 2017, an increase was noted in deaths occurring under hospice care (across home, inpatient, and nursing facility settings) and in patients’ homes (without hospice), while the proportion of deaths in hospitals and nursing homes decreased. However, the odds of death at home were lower for younger patients, female patients, and racial and ethnic minorities than they were for older patients, male patients, and white patients [4]. Racial/ethnic differences have been observed in the likelihood of dying in a hospital compared to other settings such as home, hospice, or nursing homes, with non-white individuals being more likely than non-Hispanic white individuals to die in a hospital rather than at home, in hospice, or in a nursing home [5]. The study by Kaplan et al. found that deaths at home and hospice for ESLD patients have increased overall over the past decade at the national level, with Black or African American patients experiencing the highest relative increase in hospice utilization. Black/African American patients continue to be less likely to die in hospice, suggesting that racial differences in EOL care persist despite improvements [6,7]. There has been little research on the experience with EOL care of Hispanic/Latinos patients with ESLD and hepatocellular carcinoma (HCC) [8].

While previous studies have highlighted significant state-level differences in the percentage of decedents enrolling in hospice, ESLD/HCC disease-specific variability in location of death remains unexplored [1]. Geographic factors that have been associated with higher use of hospice before death include living in wealthier areas, urban areas, areas with higher healthcare reimbursements, and areas with greater physician availability. Lower hospice utilization before death has been associated with geographic factors such as a higher number of hospital beds per capita, increased in-hospital death rates, and lower HMO enrollment [9]. An analysis of state-level variability in location of death for patients with ESLD can inform clinicians, healthcare administrators, and policy makers of the extent to which geography is a determinant of the site of EOL care. It is also unknown if the racial/ethnic differences in EOL care for these patients are consistent throughout the United States. Understanding this may elucidate interventions at the state level to improve EOL care that do not exacerbate differences between racial/ethnic groups and highlight which states may benefit more from interventions targeting specific racial/ethnic groups.

This study aimed to investigate state-level variability and racial/ethnic differences in the proportions of deaths occurring in inpatient medical facilities for patients with ESLD and HCC, with a specific focus on comparing outcomes between Non-Hispanic/Latino White (White) patients, Non-Hispanic/Latino Black/African American (Black), and Hispanic/Latino patients.

## Methods

### Maps

A retrospective cross-sectional analysis was conducted using data from the Centers for Disease Control and Prevention Wide-Ranging ONLine Data for Epidemiologic Research (CDC WONDER) Underlying Cause of Death database spanning 2018 to 2022 [10]. The proportion of patients over the age of 25 with ESLD, HCC, and both conditions who died at the decedent’s home, in hospice facilities, or in a combination of both settings was calculated for Black, White, and Hispanic/Latino patients and mapped [6]. Data for individuals identifying as American Indian or Alaska Native, Asian, Native Hawaiian or Other Pacific Islander, and more than one race was also available on CDC WONDER but not analyzed since many states did not have ESLD deaths for patients in these racial categories. Due to the nature of coding of in-home hospice in CDC WONDER data, we focused on deaths at 1) medical facility–inpatient, 2) the decedent’s home, 3) the hospice facility, and 4) decedent’s home and hospice facility combined. Other locations of death included in CDC WONDER for which data was not analyzed were: medical facility–outpatient or ER; nursing home/long-term care; other. Death attributed to ESLD was defined similarly to other studies: a) cirrhosis (K74.4, K74.5, K74.6, K70.3, K71.7); b) hepatic failure (K72.XX, K70.4, K71.7); and c) HCC (C22.0) [6]. Mapping was utilized to compare the proportions of locations of death for these categories across the US. A sensitivity analysis was performed with data spanning 2018–2019 and 2020–2021 for changes in trends of locations of death during the COVID-19 pandemic [11,12].

### Statistical Analysis

The primary outcome was the proportion of ESLD and HCC deaths occurring in inpatient medical facilities, decedent’s home, hospice facilities, and home and hospice combined, and the main predictor was the availability of hospice agencies per state. Analyses were stratified by racial/ethnic group. The proportion of ESLD and HCC deaths occurring in each location was calculated as described above. The number of hospice agencies enrolled in Medicare in each state was obtained via the Hospice Enrollments dataset from the Centers for Medicare & Medicaid Services. The number of hospice agencies per capita per 100,000 persons was calculated using the 2020 Census population results for all 50 continental states as a surrogate measure for hospice availability in each state. Spearman’s rank correlation coefficients (ρ) were calculated to assess associations between the number of hospice agencies and the proportion of home deaths by race/ethnicity. This non-parametric test was used due to the ordinal nature and non-normal distribution of state-level data. Statistical significance was set at *p* < 0.05.

## Results

### Maps

**Fig. 1:**
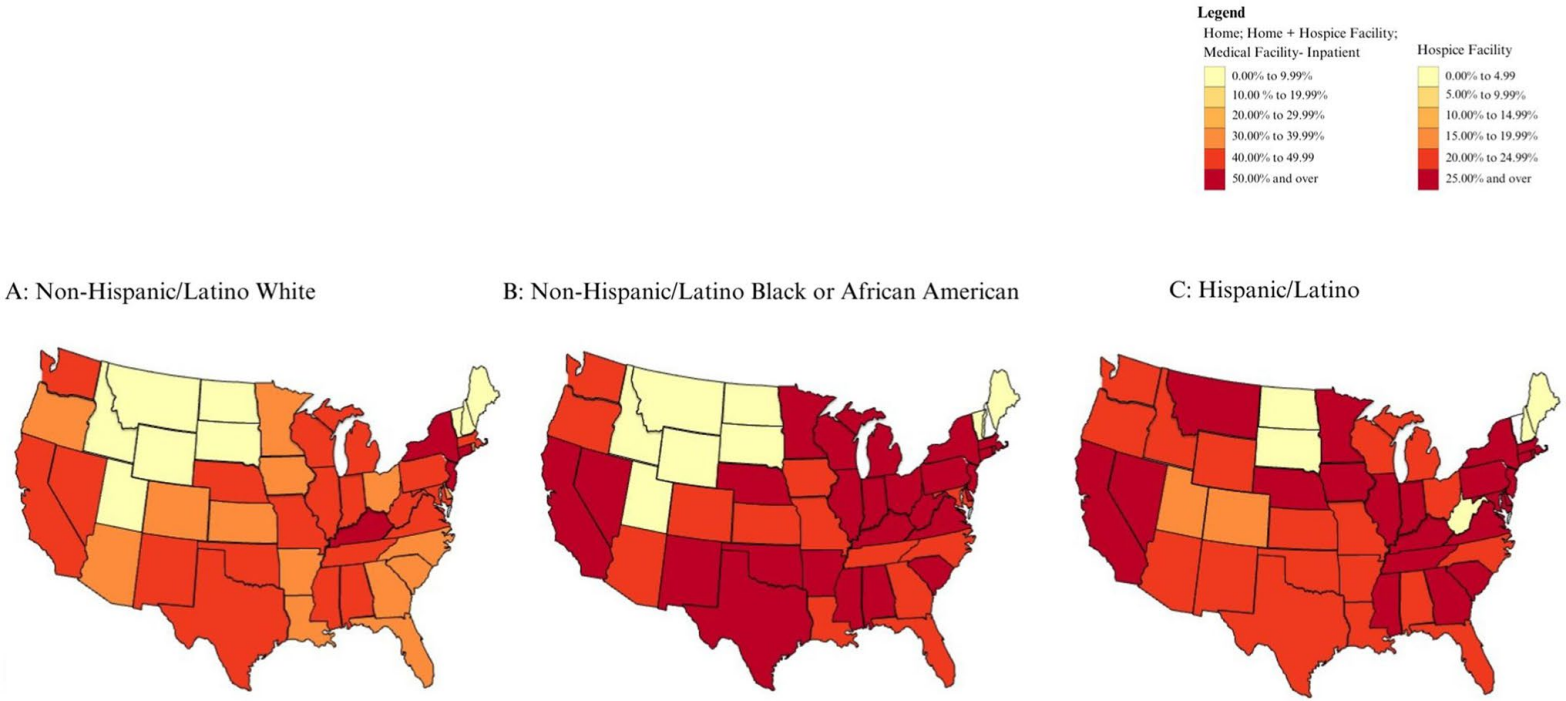
Proportion of location of death of patients with end-stage liver disease and hepatocellular carcinoma who died in a medical facility-inpatient

**Fig. 2:**
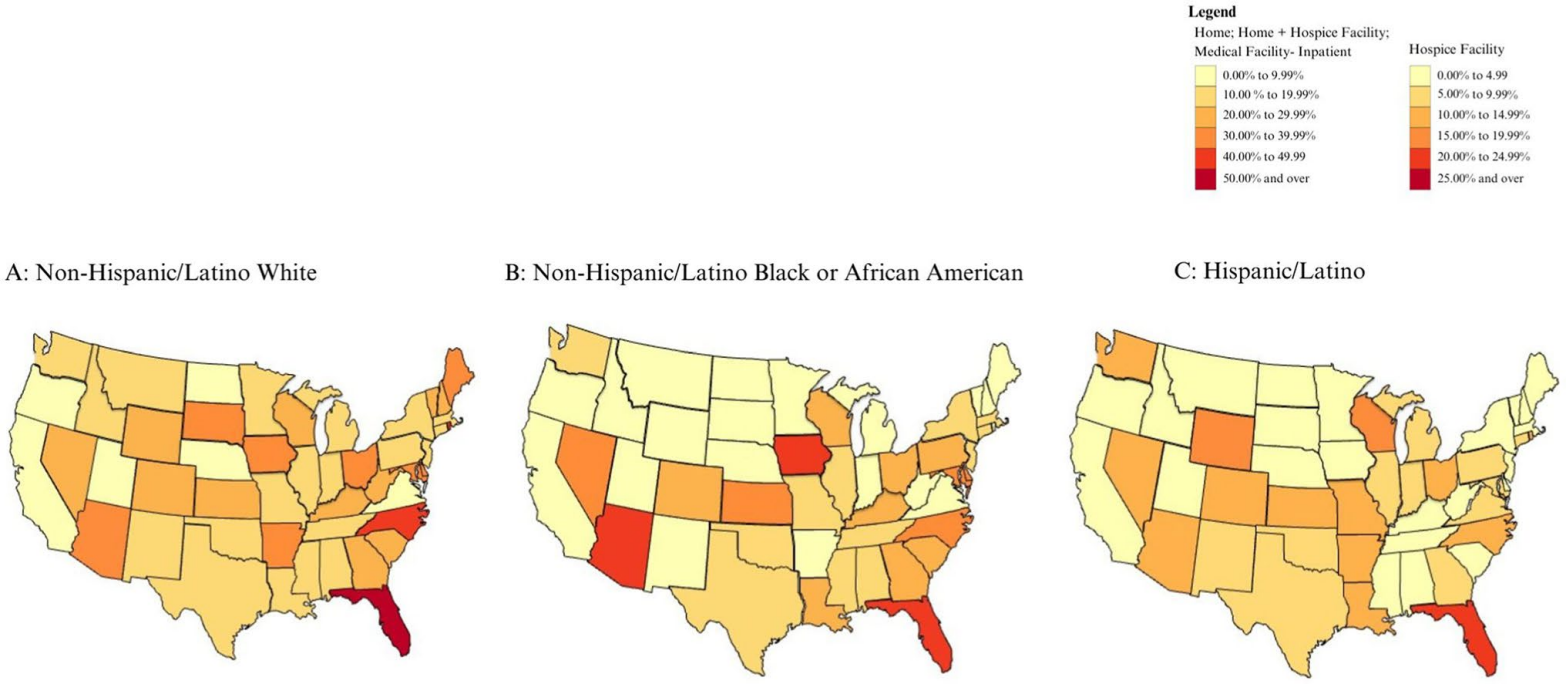
Proportion of patients with end-stage liver disease and hepatocellular carcinoma who died in a hospice facility

**Fig. 3:**
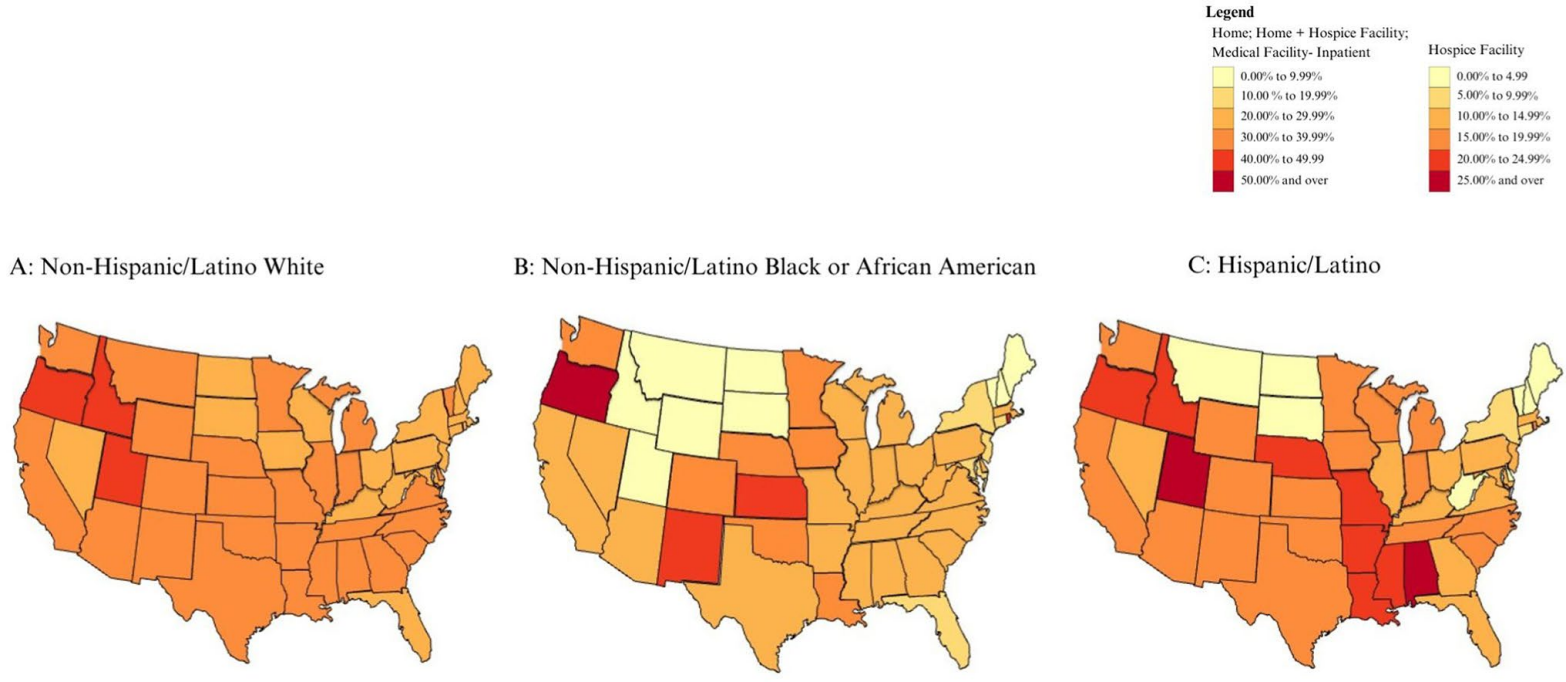
Proportion of location of death of patients with end-stage liver disease and hepatocellular carcinoma who died at decedent’s home

**Fig. 4:**
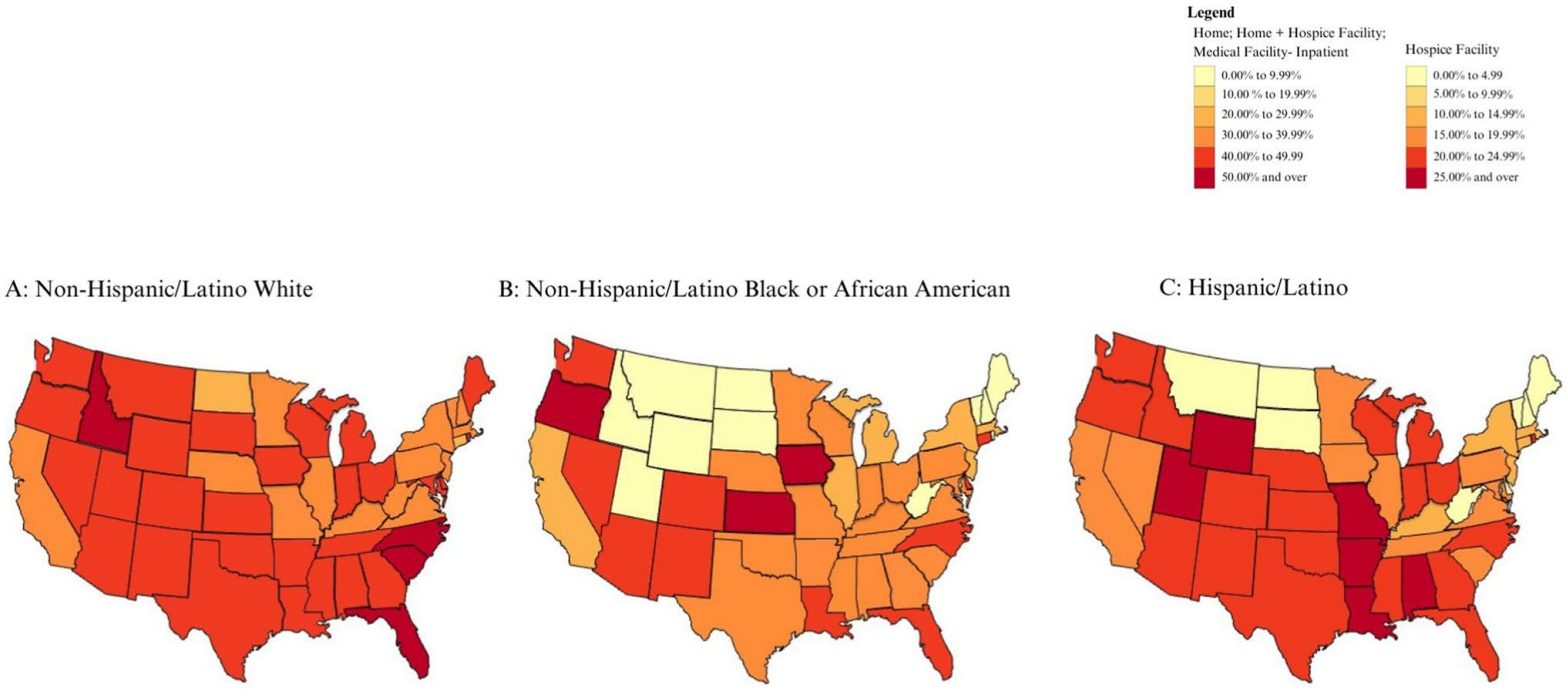
Proportion of location of death of patients with end-stage liver disease and hepatocellular carcinoma who died in a hospice facility or at decedent’s home

Variability was observed in the proportions of patients dying in inpatient medical facilities, at home, and hospice facilities across different regions of the country for all racial and ethnic groups (Figs. 1, 2, 3, and 4). White ESLD and HCC patients appeared to die in inpatient medical facilities at smaller proportions in comparison to Black and Hispanic/Latino patients (Supplemental Fig. 1). There were four states where over 50% of White ESLD and HCC decedents died in inpatient medical facilities (Connecticut, Kentucky, New Jersey, New York), compared with 22 states for Hispanic/Latino decedents and 26 states for Black decedents. There were nine states where less than 9.99% of White and Black ESLD and HCC decedents died in inpatient medical facilities (Idaho, Maine, Montana, New Hampshire, North Dakota, South Dakota, Utah, Vermont, Wyoming), compared with 6 states for Hispanic/Latino decedents (Maine, New Hampshire, North Dakota, South Dakota, Vermont, West Virginia).

The proportion of patients with ESLD or HCC who died at home or in hospice facilities was generally higher for White patients (Fig. 4). In Alabama, Arkansas, Colorado, Indiana, Kansas, Louisiana, Mississippi, Missouri, Nebraska, New Mexico, Ohio, Rhode Island, Utah, Washington, Wisconsin, and Wyoming, however, the proportion was higher for Hispanic/Latino patients than for White patients. The proportion of patients with ESLD or HCC who died at home or in hospice facilities was higher for Black patients than for White patients in Iowa, Kansas, and Oregon. Differences in the proportion of deaths at home between racial groups were more apparent and homogeneous than differences in hospice facility utilization (Figs. 2 and 3).

Hispanic/Latino patients appeared to die at home with ESLD and/or HCC in similar proportions to White patients in most states (Fig. 3A and C; Supplemental Figs. 3A and C, 7A and C) but had lower proportions of deaths in hospice facilities (Fig. 2A and C; Supplemental Figs. 2A and C, 6A and C). The proportion of ESLD patients dying in hospice and at home was higher for White patients (Supplemental Fig. 4A) than for Black patients (Supplemental Fig. 4B).

Patients with HCC exhibited the highest proportions of deaths at the decedent’s homes, with differences noted between non-Hispanic/Latino White and Black/African American patients in most of the country (Supplemental Fig. 7A and B). Hispanic/Latino patients with HCC (Supplemental Fig. 7C) died at home at similar proportions to non-Hispanic/Latino White patients with HCC (Supplemental Fig. 7A), except for states DE, GA, KY, ME, MS, MT, ND, NH, SD, VT, WV, and WY, where there were no home deaths recorded for Hispanic/Latino patients. Non-Hispanic/Latino White patients with HCC also had a higher proportion of deaths in inpatient medical facilities than non-Hispanic/Latino Black/African American and Hispanic/Latino patients (Supplemental Fig. 5).

An increase in the proportion of home deaths was observed from 2020 to 2021 compared to 2018 to 2019 in most states in the sensitivity analysis, although this did not impact our primary comparison of White, Black, and Hispanic/Latino patients.

### Secondary Analyses

Across racial/ethnic groups, mostly weak, non-statistically significant correlations were observed between the number of hospice agencies per 100,000 persons in each state and the proportion of deaths by location among ESLD and HCC patients (Table 1). Weak negative correlations with inpatient medical facility deaths were observed for White (*ρ* = –0.24, *p* = 0.09), Black (*ρ* = –0.25, *p* = 0.08), and Hispanic/Latino patients (*ρ* = –0.23, *p* = 0.11). Similarly, weak negative correlations with hospice facility deaths were found among White (*ρ* = –0.03, *p* = 0.89) and Black patients (*ρ* = –0.03, *p* = 0.86), while Hispanic/Latino patients showed a weak positive correlation (*ρ* = 0.05, *p* = 0.74).

**Table 1:** Spearman’s rank correlation between registered state hospice agencies per capita per 100,000 persons and racial/ethnic group ESLD and HCC death locations

Location of death	Race/ethnicity	Spearman’s ρ	*p*-value
	White	− 0.24	0.09
Inpatient Medical Facility	Black	− 0.25	0.08
	Hispanic/Latino	− 0.23	0.11
	White	− 0.03	0.85
Hospice	Black	− 0.03	0.86
	Hispanic/Latino	0.05	0.74
	White	0.33	**0.02**^**a**^
Home	Black	0.27	0.06
	Hispanic/Latino	0.38	**0.01**^**a**^
	White	0.17	0.24
Home and Hospice	Black	0.16	0.27
	Hispanic/Latino	0.27	0.06

^a^Indicates significant *p*-value, α = 0.05

Statistically significant moderate positive correlations were observed between the number of hospice agencies and the proportion of home deaths among White (*ρ* = 0.33, *p* = 0.02) and Hispanic/Latino patients (*ρ* = 0.38, *p* = 0.01). A marginally non-significant positive correlation was also noted for Black patients (*ρ* = 0.27, *p* = 0.06).

Weak non-significant positive correlations were observed when combining home and hospice facility deaths for White (*ρ* = 0.17, *p* = 0.24), Black (*ρ* = 0.16, *p* = 0.27), and Hispanic/Latino patients (*ρ* = 0.27, *p* = 0.06).

## Discussion

State-level data demonstrates that the location of death varies widely across the country for different racial/ethnic groups. Two novel findings revealed in the results of this study included a) the states where Black and Hispanic/Latino patients die in inpatient facilities at higher proportions than White patients; b) fewer racial/ethnic differences in the location of death are observed with HCC compared to ESLD. Findings also suggest that greater hospice availability may increase the likelihood of dying at home, notably among White and Hispanic/Latino patients, and a weaker association with a decreased likelihood of dying in inpatient medical facilities.

Hospice utilization for ESLD and HCC patients in this study differed from patterns in the general Medicare population. For instance, Utah had the highest rate of hospice use among Medicare decedents in 2022 (59.61%) and in the last six months of life in 2011 (60.8%). Yet, in our study, none of the ESLD or HCC patients in Utah—regardless of racial or ethnic group—died in a hospice facility. Across all states and racial/ethnic groups, the proportion of ESLD and HCC patients who died in hospice facilities was consistently lower than the overall Medicare population [12]. Although Black ESLD patients had the highest increase in the adoption of hospice services from 2003 to 2018 and Hispanic Medicare beneficiaries had the highest increase in 2022, our analysis reveals that both groups continue to be less likely to die at home or in a hospice facility independent of state-level variances when compared to White patients [6,12]. Concurrently, White patients with ESLD had lower proportions of deaths in inpatient medical facilities. These findings are consistent with studies demonstrating that Black/African American patients tend to receive more aggressive treatments than White patients at the end of life, with higher rates of multiple emergency room visits and hospitalizations, especially in noncancer patients [13]. They are also consistent with studies highlighting lower hospice enrollment rates for Hispanic/Latino patients [8]. Like a study on geographic variation of hospice use analyzing Medicare data for patients with all conditions, there was no correlation between states with high vs. low in hospice deaths and the proportion of deaths in inpatient medical facilities [9].

Currently, there is no established benchmark for the appropriate proportion of ESLD deaths that should occur in hospice versus inpatient medical settings. Hospice enrollment criteria require that patients have a prognosis of 6 months or less; however, long-standing concerns have existed regarding clinical guidelines for determining prognosis in noncancer diseases. A study found that recommended clinical prediction criteria for ESLD, chronic obstructive pulmonary disease, and heart failure were effective in excluding patients who lived longer than 6 months from hospice enrollment, but simultaneously excluded the majority of patients who were dead in 6 months or less, thus excluding the very patients they were supposed to identify [14]. As a result, we cannot determine how far the observed racial and ethnic disparities in the location of death deviate from an ideal standard of how many patients would die in hospice instead of inpatient medical facilities. Moreover, it may be inappropriate to assume that dying at home or in a hospice facility always reflects high-quality EOL care.

However, deaths in inpatient medical facilities are generally undesirable as they incur higher healthcare costs, are often not in line with the decedent’s preferences, can result in worse psychological outcomes for next of kin, and are associated with unmet patient needs and poor palliation of symptoms [4,15]. Liver transplantation is curative for patients with ESLD, yet many patients are unable to undergo it. The curative nature of liver transplantation may also result in delays to the initiation of hospice care [16]. More work is necessary to establish ideal hospice referral criteria for patients with ESLD, similar to the well-established pathways for cancer patients. Current ESLD hospice referral criteria for Medicare beneficiaries do not include the Model for End-stage Liver Disease (MELD) score, the primary prognostic factor utilized for patients with ESLD. The criteria do include other outdated and less clinically relevant factors. Liver transplant criteria are not included in Medicare hospice referral criteria, a significant limitation given that patients would forego the option of curative transplant treatment if choosing to enroll in hospice care [2]. The existence of more appropriate guidelines for cancer patients compared to ESLD patients may help explain why racial and ethnic disparities in location of death were less pronounced among patients with HCC compared to those with ESLD. A previous study comparing cancer and noncancer deaths found that ESLD patients who enroll in hospice are more likely to have HCC and a greater comorbidity burden compared to those who do not, and referrals are more likely to have been made by an oncologist [2]. When ESLD and HCC were mapped separately in our study, ESLD patients appeared to have a higher proportion of deaths occurring at a hospice facility than HCC patients (Supplemental Figs. 2 and 7), but HCC patients had a higher proportion of deaths at home (Supplemental Figs. 3 and 7). However, it is unknown which patients coded as ESLD or HCC as the cause of death on CDC WONDER had a concurring condition.

Preferences for hospice by different racial/ethnic groups should also be explored, but the variability in different states in locations of death for all racial and ethnic groups analyzed in this study, as well as another study examining various chronic conditions, indicates that patient preferences and cultural factors alone do not account for low hospice utilization by Black and Hispanic/Latino patients [9].

The availability of hospice agencies in each state, measured by the number of registered hospice agencies per capita per 100,000 persons, may be a possible state-level factor influencing the differences in the proportion of deaths occurring in inpatient medical facilities over home and hospice by state. Across all racial/ethnic categories, there was a non-significant but consistent negative correlation between the proportion of deaths occurring in inpatient medical facilities and hospice agency availability. In contrast, a significant moderate positive correlation was observed between the proportion of deaths occurring at home and hospice agency availability. These findings align with the hypothesis that expanding hospice access may reduce the proportion of deaths occurring in inpatient medical facilities that are often considered less desirable and increase the likelihood of death at the decedent’s home. A limitation to the use of the number of hospice agencies registered in a state per capita as a measure of hospice availability is the assumption that all hospice agencies serve an equal number of patients. The number of registered hospice agencies in each state is also not indicative of accessibility to quality hospice care at the end of life for all decedents.

Another state-level factor possibly influencing the variability in proportions of location of death is the presence of laws pertaining to palliative care in a state. A study aiming to determine whether US state palliative care legislation is associated with place of death from cancer found that decedents in states with palliative care laws had an increased likelihood of dying at home or in hospice. This study included any legislation pertaining to palliative care and end of life, including those they defined as “prescriptive” and required clinicians to take certain action if patients met criteria for palliative care, as well as non-prescriptive, such as the establishment of an advisory council to investigate palliative care practices in a given state. Their secondary analysis of decedents of noncancer serious illnesses, which included liver disease, also found a positive association between the presence of palliative care legislation and dying at home or in hospice [17].

A strength of this study is that the utilization of the CDC WONDER database allows for the inclusion of non-Medicare beneficiaries, as a large number of studies regarding EOL care focus on the Medicare-insured population [18]. A significant limitation is that our study analyzed deaths at the decedent’s home, hospice facilities, and both combined since we could not determine from the CDC WONDER data which deaths at home may have also been hospice deaths. The data on hospice facility deaths alone may have underestimated the proportion of ESLD and HCC patients who had died under a hospice admission while at the decedent’s home, nursing home, etc. Deaths occurring at home without hospice care may have taken place under conditions that do not align with a good quality of death. Another limitation to the interpretation of results is that states such as Kansas and Iowa, where Black/African American patients had higher rates of deaths at home and hospice facilities (Fig. 3A and B), also have smaller populations of Black/African American people, possibly contributing to the departure from the general trend in differences. We are unable to determine important EOL quality measures from the data beyond the location of death, including duration of stay in inpatient medical facilities or hospice and transitions of location of care prior to death. The majority of patients with cirrhosis who enroll in hospice have been found to have late enrollments, with a median of 9 days before death.^2^ The use of measures such as the Area Deprivation Index (ADI) and Social Vulnerability Index (SVI) that examine factors contributing to regional variability in the location of death was not feasible, as these indices are measured at the ZIP code level. Analyses of racial and ethnic differences in the location of death by ZIP code would have limited statistical power due to the small number of ESLD and HCC decedents in each racial/ethnic group within each ZIP code.

Findings from this study identify states where policies and programs that reduce inpatient deaths for ESLD patients may be most needed. Targeted interventions to improve access to high-quality EOL care for all patients address the national variability of hospice use, especially for those who are Black or African American and in states with high numbers of inpatient deaths and low rates of hospice use, should also be identified and implemented. These may include increasing the number of registered hospice agencies per capita as well as adding palliative care laws to state legislatures. Surrogate measures of state-level hospice accessibility and quality of EOL care should be further developed for use in future research on factors contributing to interstate variability and racial differences in EOL care for patients with ESLD and HCC.

During the preparation of this work, the authors used ChatGPT in order to improve language and readability. After using this tool/service, the authors reviewed and edited the content as needed and took full responsibility for the content of the publication.

## Supplementary Information

Below is the link to the electronic supplementary material.

Supplementary file1 (ZIP 1382 KB)

## Data Availability

Data collected for analysis for this study can be found in CDC Wonder Underlying Causes of Death https://wonder.cdc.gov/deaths-by-underlying-cause.html and the Hospice Enrollments dataset from the Centers for Medicare and Medicaid Services https://data.cms.gov/provider-characteristics/hospitals-and-other-facilities/hospice-enrollments.
