# Supplementary material for: State-Level Variability in Location of Death of Patients with End-Stage Liver Disease
Source: Dig Dis Sci. 2025 Oct 8;71(3):933–40. doi: 10.1007/s10620-025-09433-w (PMC12982227; doi:10.1007/s10620-025-09433-w)
Supplement: Supplementary file 1 — Supplementary file1 (ZIP 1382 KB) [file 10620_2025_9433_MOESM1_ESM.zip › Supplementary/SDC Table 1.docx]

**Table 1**

*Proportion of Patients With End-Stage Liver Disease and Hepatocellular Carcinoma Who Died in a Hospice Facility*

| **State** | **Non- Hispanic/Latino White** | **Non- Hispanic/Latino Black or African American** | **Hispanic/Latino** |
| --- | --- | --- | --- |
| Alabama | 7.9 | 6.6 | 0.0 |
| Alaska | 0.0 | 0.0 | 0.0 |
| Arizona | 15.2 | 17.7 | 10.9 |
| Arkansas | 17.4 | 4.4 | 14.5 |
| California | 2.4 | 2.5 | 1.8 |
| Colorado | 12.8 | 14.9 | 12.3 |
| Connecticut | 5.3 | 6.8 | 6.1 |
| Delaware | 18.8 | 18.1 | 0.0 |
| District of Columbia | 0.0 | 9.3 | 0.0 |
| Florida | 30.0 | 20.6 | 22.0 |
| Georgia | 12.8 | 13.8 | 9.9 |
| Hawaii | 12.8 | 0.0 | 0.0 |
| Idaho | 7.2 | 0.0 | 0.0 |
| Illinois | 7.0 | 6.5 | 7.4 |
| Indiana | 7.3 | 4.7 | 8.3 |
| Iowa | 15.9 | 23.3 | 0.0 |
| Kansas | 13.3 | 16.1 | 12.9 |
| Kentucky | 12.5 | 11.0 | 0.0 |
| Louisiana | 9.8 | 10.9 | 13.1 |
| Maine | 17.4 | 0.0 | 0.0 |
| Maryland | 16.9 | 17.8 | 7.7 |
| Massachusetts | 7.6 | 3.6 | 4.7 |
| Michigan | 8.0 | 4.5 | 5.6 |
| Minnesota | 5.4 | 0.0 | 0.0 |
| Mississippi | 9.4 | 5.9 | 0.0 |
| Missouri | 5.4 | 7.8 | 13.9 |
| Montana | 6.7 | 0.0 | 0.0 |
| Nebraska | 4.0 | 0.0 | 0.0 |
| Nevada | 14.4 | 17.5 | 13.5 |
| New Hampshire | 10.5 | 0.0 | 0.0 |
| New Jersey | 6.9 | 5.2 | 3.7 |
| New Mexico | 5.5 | 0.0 | 6.4 |
| New York | 8.8 | 5.5 | 4.8 |
| North Carolina | 20.4 | 16.7 | 13.5 |
| North Dakota | 0.0 | 0.0 | 0.0 |
| Ohio | 16.2 | 11.5 | 18.0 |
| Oklahoma | 6.1 | 5.0 | 5.9 |
| Oregon | 4.0 | 0.0 | 0.0 |
| Pennsylvania | 8.4 | 11.1 | 7.9 |
| Rhode Island | 20.8 | 0.0 | 18.6 |
| South Carolina | 14.0 | 12.9 | 0.0 |
| South Dakota | 16.9 | 0.0 | 0.0 |
| Tennessee | 5.5 | 8.9 | 0.0 |
| Texas | 9.7 | 9.2 | 6.6 |
| Utah | 0.0 | 0.0 | 0.0 |
| Vermont | 9.5 | 0.0 | 0.0 |
| Virginia | 4.6 | 4.4 | 6.2 |
| Washington | 9.5 | 8.1 | 10.3 |
| West Virginia | 12.0 | 0.0 | 0.0 |
| Wisconsin | 11.5 | 14.6 | 15.0 |
| Wyoming | 14.2 | 0.0 | 19.7 |
