# Supplementary material for: State-Level Variability in Location of Death of Patients with End-Stage Liver Disease
Source: Dig Dis Sci. 2025 Oct 8;71(3):933–40. doi: 10.1007/s10620-025-09433-w (PMC12982227; doi:10.1007/s10620-025-09433-w)
Supplement: Supplementary file 1 — Supplementary file1 (ZIP 1382 KB) [file 10620_2025_9433_MOESM1_ESM.zip › Supplementary/SDC Table 10.docx]

**Table 10**

*Proportion of Location of Death of Patients With Hepatocellular Carcinoma Who Died at Decedent's Home*

| **State** | **Non- Hispanic/Latino White** | **Non- Hispanic/Latino Black/African American** | **Hispanic/Latino** |
| --- | --- | --- | --- |
| Alabama | 64.4 | 42.1 | 0.0 |
| Alaska | 65.8 | 0.0 | 0.0 |
| Arizona | 46.9 | 38.3 | 59.9 |
| Arkansas | 44.4 | 41.5 | 100.0 |
| California | 54.5 | 38.4 | 55.2 |
| Colorado | 51.0 | 46.0 | 59.0 |
| Connecticut | 44.9 | 40.0 | 37.7 |
| Delaware | 43.4 | 44.1 | 0.0 |
| District of Columbia | 100.0 | 25.0 | 0.0 |
| Florida | 36.8 | 28.8 | 35.6 |
| Georgia | 53.6 | 38.8 | 57.6 |
| Hawaii | 68.2 | 0.0 | 0.0 |
| Idaho | 60.2 | 0.0 | 100.0 |
| Illinois | 51.1 | 32.9 | 50.2 |
| Indiana | 51.2 | 43.2 | 69.8 |
| Iowa | 40.5 | 52.4 | 0.0 |
| Kansas | 42.9 | 100.0 | 64.5 |
| Kentucky | 43.8 | 41.9 | 0.0 |
| Louisiana | 52.6 | 38.4 | 100.0 |
| Maine | 40.9 | 0.0 | 0.0 |
| Maryland | 46.2 | 27.7 | 57.6 |
| Massachusetts | 48.2 | 40.5 | 45.7 |
| Michigan | 56.8 | 39.0 | 77.0 |
| Minnesota | 49.8 | 45.2 | 100.0 |
| Mississippi | 41.9 | 25.6 | 0.0 |
| Missouri | 56.7 | 41.8 | 100.0 |
| Montana | 54.1 | 0.0 | 0.0 |
| Nebraska | 53.8 | 0.0 | 100.0 |
| Nevada | 49.8 | 27.9 | 55.6 |
| New Hampshire | 50.2 | 0.0 | 0.0 |
| New Jersey | 43.6 | 25.7 | 37.6 |
| New Mexico | 54.5 | 0.0 | 56.4 |
| New York | 44.2 | 22.9 | 26.8 |
| North Carolina | 45.2 | 35.3 | 78.8 |
| North Dakota | 37.4 | 0.0 | 0.0 |
| Ohio | 43.4 | 30.8 | 38.5 |
| Oklahoma | 53.5 | 46.3 | 100.0 |
| Oregon | 59.2 | 51.7 | 74.6 |
| Pennsylvania | 42.8 | 34.8 | 48.3 |
| Rhode Island | 40.3 | 0.0 | 100.0 |
| South Carolina | 51.8 | 33.2 | 100.0 |
| South Dakota | 36.9 | 0.0 | 0.0 |
| Tennessee | 56.4 | 39.5 | 100.0 |
| Texas | 49.1 | 35.0 | 54.0 |
| Utah | 68.4 | 0.0 | 100.0 |
| Vermont | 55.8 | 0.0 | 0.0 |
| Virginia | 55.7 | 47.1 | 51.1 |
| Washington | 49.9 | 59.5 | 64.4 |
| West Virginia | 53.3 | 0.0 | 0.0 |
| Wisconsin | 44.4 | 37.4 | 71.4 |
| Wyoming | 56.0 | 0.0 | 0.0 |
