# Supplementary material for: State-Level Variability in Location of Death of Patients with End-Stage Liver Disease
Source: Dig Dis Sci. 2025 Oct 8;71(3):933–40. doi: 10.1007/s10620-025-09433-w (PMC12982227; doi:10.1007/s10620-025-09433-w)
Supplement: Supplementary file 1 — Supplementary file1 (ZIP 1382 KB) [file 10620_2025_9433_MOESM1_ESM.zip › Supplementary/SDC Table 11.docx]

**Table 11**

*Proportion of Location of Death of Patients With Hepatocellular Carcinoma Who Died in a Hospice Facility or at Decedent's Home*

| **State** | **Non- Hispanic/Latino White** | **Non- Hispanic/Latino Black/African American** | **Hispanic/Latino** |
| --- | --- | --- | --- |
| Alabama | 73.7 | 53.3 | 0.0 |
| Alaska | 65.8 | 0.0 | 0.0 |
| Arizona | 69.4 | 68.1 | 74.4 |
| Arkansas | 66.9 | 41.5 | 100.0 |
| California | 58.9 | 43.0 | 58.9 |
| Colorado | 70.2 | 68.0 | 80.8 |
| Connecticut | 55.1 | 40.0 | 37.7 |
| Delaware | 73.8 | 44.1 | 0.0 |
| District of Columbia | 100.0 | 39.6 | 0.0 |
| Florida | 72.5 | 55.8 | 62.9 |
| Georgia | 68.5 | 59.3 | 76.3 |
| Hawaii | 68.2 | 0.0 | 0.0 |
| Idaho | 69.3 | 0.0 | 100.0 |
| Illinois | 60.2 | 41.2 | 62.7 |
| Indiana | 61.1 | 52.3 | 69.8 |
| Iowa | 62.0 | 52.4 | 0.0 |
| Kansas | 59.6 | 100.0 | 64.5 |
| Kentucky | 62.8 | 61.3 | 0.0 |
| Louisiana | 64.5 | 51.7 | 100.0 |
| Maine | 68.9 | 0.0 | 0.0 |
| Maryland | 70.3 | 56.5 | 57.6 |
| Massachusetts | 60.6 | 40.5 | 59.0 |
| Michigan | 67.6 | 46.3 | 77.0 |
| Minnesota | 58.4 | 45.2 | 100.0 |
| Mississippi | 59.2 | 44.4 | 0.0 |
| Missouri | 65.3 | 55.5 | 100.0 |
| Montana | 60.1 | 0.0 | 0.0 |
| Nebraska | 53.8 | 0.0 | 100.0 |
| Nevada | 67.1 | 58.1 | 70.8 |
| New Hampshire | 68.0 | 0.0 | 0.0 |
| New Jersey | 53.6 | 33.2 | 47.1 |
| New Mexico | 63.5 | 0.0 | 68.7 |
| New York | 57.3 | 33.0 | 35.0 |
| North Carolina | 73.2 | 60.0 | 78.8 |
| North Dakota | 37.4 | 0.0 | 0.0 |
| Ohio | 65.8 | 48.4 | 67.3 |
| Oklahoma | 63.0 | 46.3 | 100.0 |
| Oregon | 63.5 | 51.7 | 74.6 |
| Pennsylvania | 56.6 | 52.5 | 61.5 |
| Rhode Island | 74.2 | 0.0 | 100.0 |
| South Carolina | 68.9 | 52.7 | 100.0 |
| South Dakota | 57.7 | 0.0 | 0.0 |
| Tennessee | 63.0 | 51.3 | 100.0 |
| Texas | 61.1 | 47.1 | 61.9 |
| Utah | 68.4 | 0.0 | 100.0 |
| Vermont | 74.2 | 0.0 | 0.0 |
| Virginia | 62.5 | 52.9 | 51.1 |
| Washington | 63.8 | 59.5 | 79.7 |
| West Virginia | 68.1 | 0.0 | 0.0 |
| Wisconsin | 62.0 | 65.7 | 71.4 |
| Wyoming | 70.0 | 0.0 | 0.0 |
