# Supplementary material for: State-Level Variability in Location of Death of Patients with End-Stage Liver Disease
Source: Dig Dis Sci. 2025 Oct 8;71(3):933–40. doi: 10.1007/s10620-025-09433-w (PMC12982227; doi:10.1007/s10620-025-09433-w)
Supplement: Supplementary file 1 — Supplementary file1 (ZIP 1382 KB) [file 10620_2025_9433_MOESM1_ESM.zip › Supplementary/SDC Table 12.docx]

**Table 12**

*Proportion of Patients With Hepatocellular Carcinoma Who Died in a Medical Facility- Inpatient*

| **State** | **Non- Hispanic/Latino White** | **Non- Hispanic/Latino Black/African American** | **Hispanic/Latino** |
| --- | --- | --- | --- |
| Alabama | 20.2 | 33.5 | 0.0 |
| Alaska | 34.2 | 0.0 | 0.0 |
| Arizona | 16.1 | 31.9 | 16.4 |
| Arkansas | 23.2 | 58.5 | 0.0 |
| California | 26.3 | 35.0 | 28.1 |
| Colorado | 16.5 | 32.0 | 19.2 |
| Connecticut | 41.7 | 60.0 | 62.3 |
| Delaware | 26.2 | 55.9 | 0.0 |
| District of Columbia | 0.0 | 49.3 | 0.0 |
| Florida | 19.6 | 30.9 | 29.0 |
| Georgia | 20.4 | 28.6 | 23.7 |
| Hawaii | 31.8 | 0.0 | 0.0 |
| Idaho | 13.9 | 0.0 | 0.0 |
| Illinois | 25.0 | 37.9 | 31.7 |
| Indiana | 23.1 | 39.4 | 30.2 |
| Iowa | 20.8 | 47.6 | 0.0 |
| Kansas | 25.2 | 0.0 | 35.5 |
| Kentucky | 29.6 | 38.7 | 0.0 |
| Louisiana | 21.9 | 30.7 | 0.0 |
| Maine | 20.0 | 0.0 | 0.0 |
| Maryland | 19.4 | 27.9 | 42.4 |
| Massachusetts | 25.7 | 59.5 | 30.5 |
| Michigan | 21.9 | 33.7 | 23.0 |
| Minnesota | 19.1 | 38.7 | 0.0 |
| Mississippi | 36.6 | 55.6 | 0.0 |
| Missouri | 19.2 | 33.5 | 0.0 |
| Montana | 24.0 | 0.0 | 0.0 |
| Nebraska | 22.1 | 0.0 | 0.0 |
| Nevada | 24.3 | 41.9 | 29.2 |
| New Hampshire | 24.1 | 0.0 | 0.0 |
| New Jersey | 32.5 | 55.1 | 37.6 |
| New Mexico | 20.8 | 0.0 | 20.8 |
| New York | 39.7 | 61.3 | 59.8 |
| North Carolina | 16.6 | 31.7 | 21.2 |
| North Dakota | 38.5 | 0.0 | 0.0 |
| Ohio | 19.5 | 33.8 | 32.7 |
| Oklahoma | 23.6 | 53.7 | 0.0 |
| Oregon | 21.8 | 48.3 | 25.4 |
| Pennsylvania | 28.5 | 34.8 | 30.1 |
| Rhode Island | 16.8 | 0.0 | 0.0 |
| South Carolina | 22.5 | 40.1 | 0.0 |
| South Dakota | 29.2 | 0.0 | 0.0 |
| Tennessee | 22.4 | 29.9 | 0.0 |
| Texas | 22.5 | 36.3 | 24.1 |
| Utah | 10.4 | 0.0 | 0.0 |
| Vermont | 25.8 | 0.0 | 0.0 |
| Virginia | 26.7 | 38.8 | 48.9 |
| Washington | 24.0 | 40.5 | 20.3 |
| West Virginia | 20.7 | 0.0 | 0.0 |
| Wisconsin | 19.6 | 34.3 | 28.6 |
| Wyoming | 20.0 | 0.0 | 0.0 |
