# Supplementary material for: State-Level Variability in Location of Death of Patients with End-Stage Liver Disease
Source: Dig Dis Sci. 2025 Oct 8;71(3):933–40. doi: 10.1007/s10620-025-09433-w (PMC12982227; doi:10.1007/s10620-025-09433-w)
Supplement: Supplementary file 1 — Supplementary file1 (ZIP 1382 KB) [file 10620_2025_9433_MOESM1_ESM.zip › Supplementary/SDC Table 2.docx]

**Table 2**

*Proportion of Location of Death of Patients With End-Stage Liver Disease and Hepatocellular Carcinoma Who Died at Decedent's Home*

| **State** | **Non- Hispanic/Latino White** | **Non- Hispanic/Latino Black or African American** | **Hispanic/Latino** |
| --- | --- | --- | --- |
| Alabama | 38.6 | 27.6 | 54.3 |
| Alaska | 37.8 | 0.0 | 0.0 |
| Arizona | 34.4 | 28.7 | 34.8 |
| Arkansas | 30.7 | 25.9 | 42.2 |
| California | 35.8 | 26.6 | 32.3 |
| Colorado | 33.7 | 34.6 | 37.0 |
| Connecticut | 24.3 | 20.4 | 18.7 |
| Delaware | 30.3 | 26.1 | 0.0 |
| District of Columbia | 38.6 | 14.5 | 0.0 |
| Florida | 26.1 | 19.9 | 25.1 |
| Georgia | 35.7 | 25.7 | 31.4 |
| Hawaii | 32.6 | 0.0 | 64.0 |
| Idaho | 43.0 | 0.0 | 45.2 |
| Illinois | 31.6 | 20.8 | 27.3 |
| Indiana | 33.4 | 27.1 | 35.7 |
| Iowa | 28.6 | 32.9 | 30.6 |
| Kansas | 31.6 | 40.3 | 34.4 |
| Kentucky | 26.6 | 23.0 | 24.4 |
| Louisiana | 38.9 | 30.3 | 44.4 |
| Maine | 27.9 | 0.0 | 0.0 |
| Maryland | 28.2 | 19.9 | 27.3 |
| Massachusetts | 26.4 | 23.5 | 25.1 |
| Michigan | 34.6 | 24.7 | 36.4 |
| Minnesota | 33.9 | 35.8 | 32.7 |
| Mississippi | 35.2 | 26.1 | 46.2 |
| Missouri | 34.6 | 27.5 | 40.2 |
| Montana | 34.1 | 0.0 | 0.0 |
| Nebraska | 30.7 | 31.3 | 46.5 |
| Nevada | 29.3 | 23.7 | 25.3 |
| New Hampshire | 29.4 | 0.0 | 0.0 |
| New Jersey | 28.4 | 18.3 | 21.7 |
| New Mexico | 34.6 | 45.9 | 38.1 |
| New York | 27.7 | 14.6 | 18.1 |
| North Carolina | 30.5 | 25.2 | 34.6 |
| North Dakota | 28.3 | 0.0 | 0.0 |
| Ohio | 26.5 | 22.0 | 25.8 |
| Oklahoma | 36.5 | 30.9 | 35.8 |
| Oregon | 43.1 | 55.2 | 46.4 |
| Pennsylvania | 29.8 | 24.7 | 28.1 |
| Rhode Island | 27.7 | 42.3 | 30.5 |
| South Carolina | 37.5 | 26.2 | 37.0 |
| South Dakota | 26.6 | 0.0 | 0.0 |
| Tennessee | 36.3 | 26.7 | 31.0 |
| Texas | 33.3 | 25.8 | 36.3 |
| Utah | 43.3 | 0.0 | 52.0 |
| Vermont | 30.4 | 0.0 | 0.0 |
| Virginia | 35.0 | 28.5 | 29.7 |
| Washington | 35.3 | 34.8 | 37.3 |
| West Virginia | 27.7 | 20.4 | 0.0 |
| Wisconsin | 29.9 | 22.1 | 31.8 |
| Wyoming | 32.2 | 0.0 | 34.4 |
