# Supplementary material for: State-Level Variability in Location of Death of Patients with End-Stage Liver Disease
Source: Dig Dis Sci. 2025 Oct 8;71(3):933–40. doi: 10.1007/s10620-025-09433-w (PMC12982227; doi:10.1007/s10620-025-09433-w)
Supplement: Supplementary file 1 — Supplementary file1 (ZIP 1382 KB) [file 10620_2025_9433_MOESM1_ESM.zip › Supplementary/SDC Table 3.docx]

**Table 3**

*Proportion of Location of Death of Patients With End-Stage Liver Disease and Hepatocellular Carcinoma Who Died in a Hospice Facility or at Decedent's Home*

| **State** | **Non- Hispanic/Latino White** | **Non- Hispanic/Latino Black or African American** | **Hispanic/Latino** |
| --- | --- | --- | --- |
| Alabama | 46.5 | 34.2 | 54.3 |
| Alaska | 37.8 | 0.0 | 0.0 |
| Arizona | 49.5 | 46.3 | 45.7 |
| Arkansas | 48.1 | 30.2 | 56.6 |
| California | 38.3 | 29.1 | 34.1 |
| Colorado | 46.5 | 49.5 | 49.4 |
| Connecticut | 29.7 | 27.2 | 24.8 |
| Delaware | 49.1 | 44.2 | 0.0 |
| District of Columbia | 38.6 | 23.8 | 0.0 |
| Florida | 56.1 | 40.5 | 47.1 |
| Georgia | 48.5 | 39.6 | 41.3 |
| Hawaii | 45.4 | 0.0 | 64.0 |
| Idaho | 50.1 | 0.0 | 45.2 |
| Illinois | 38.6 | 27.3 | 34.7 |
| Indiana | 40.6 | 31.8 | 44.0 |
| Iowa | 44.5 | 56.2 | 30.6 |
| Kansas | 44.9 | 56.5 | 47.3 |
| Kentucky | 39.1 | 34.0 | 24.4 |
| Louisiana | 48.7 | 41.2 | 57.6 |
| Maine | 45.2 | 0.0 | 0.0 |
| Maryland | 45.1 | 37.7 | 35.0 |
| Massachusetts | 34.0 | 27.1 | 29.8 |
| Michigan | 42.6 | 29.2 | 42.0 |
| Minnesota | 39.3 | 35.8 | 32.7 |
| Mississippi | 44.6 | 32.0 | 46.2 |
| Missouri | 39.9 | 35.3 | 54.1 |
| Montana | 40.8 | 0.0 | 0.0 |
| Nebraska | 34.7 | 31.3 | 46.5 |
| Nevada | 43.7 | 41.2 | 38.8 |
| New Hampshire | 39.9 | 0.0 | 0.0 |
| New Jersey | 35.3 | 23.5 | 25.3 |
| New Mexico | 40.1 | 45.9 | 44.5 |
| New York | 36.5 | 20.1 | 22.9 |
| North Carolina | 50.8 | 41.9 | 48.0 |
| North Dakota | 28.3 | 0.0 | 0.0 |
| Ohio | 42.7 | 33.5 | 43.8 |
| Oklahoma | 42.6 | 35.9 | 41.7 |
| Oregon | 47.1 | 55.2 | 46.4 |
| Pennsylvania | 38.2 | 35.9 | 36.0 |
| Rhode Island | 48.5 | 42.3 | 49.2 |
| South Carolina | 51.4 | 39.1 | 37.0 |
| South Dakota | 43.5 | 0.0 | 0.0 |
| Tennessee | 41.8 | 35.5 | 31.0 |
| Texas | 43.0 | 34.9 | 42.9 |
| Utah | 43.3 | 0.0 | 52.0 |
| Vermont | 39.8 | 0.0 | 0.0 |
| Virginia | 39.6 | 32.9 | 35.9 |
| Washington | 44.8 | 42.9 | 47.6 |
| West Virginia | 39.8 | 0.0 | 0.0 |
| Wisconsin | 41.4 | 36.8 | 46.8 |
| Wyoming | 46.5 | 0.0 | 54.1 |
