# Supplementary material for: State-Level Variability in Location of Death of Patients with End-Stage Liver Disease
Source: Dig Dis Sci. 2025 Oct 8;71(3):933–40. doi: 10.1007/s10620-025-09433-w (PMC12982227; doi:10.1007/s10620-025-09433-w)
Supplement: Supplementary file 1 — Supplementary file1 (ZIP 1382 KB) [file 10620_2025_9433_MOESM1_ESM.zip › Supplementary/SDC Table 4.docx]

**Table 4**

*Proportion of Patients With End-Stage Liver Disease and Hepatocellular Carcinoma Who Died in a Medical Facility- Inpatient*

| **State** | **Non- Hispanic/Latino White** | **Non- Hispanic/Latino Black or African American** | **Hispanic/Latino** |
| --- | --- | --- | --- |
| Alabama | 43.9 | 54.9 | 45.7 |
| Alaska | 0.0 | 0.0 | 100.0 |
| Arizona | 33.8 | 47.6 | 40.8 |
| Arkansas | 38.4 | 58.6 | 43.4 |
| California | 44.4 | 52.9 | 52.1 |
| Colorado | 38.6 | 41.5 | 38.8 |
| Connecticut | 57.4 | 62.8 | 63.7 |
| Delaware | 39.4 | 55.8 | 100.0 |
| District of Columbia | 61.4 | 67.7 | 100.0 |
| Florida | 33.5 | 47.7 | 43.9 |
| Georgia | 38.7 | 49.4 | 53.6 |
| Hawaii | 0.0 | 0.0 | 36.0 |
| Idaho | 0.0 | 0.0 | 42.1 |
| Illinois | 43.4 | 58.1 | 53.0 |
| Indiana | 42.5 | 55.5 | 50.2 |
| Iowa | 37.7 | 43.8 | 69.4 |
| Kansas | 37.3 | 43.5 | 45.5 |
| Kentucky | 50.1 | 56.4 | 75.6 |
| Louisiana | 34.8 | 42.6 | 42.4 |
| Maine | 0.0 | 0.0 | 0.0 |
| Maryland | 41.1 | 49.0 | 60.5 |
| Massachusetts | 49.3 | 60.6 | 56.5 |
| Michigan | 44.0 | 55.3 | 45.9 |
| Minnesota | 38.7 | 51.1 | 51.8 |
| Mississippi | 42.4 | 57.1 | 53.8 |
| Missouri | 40.4 | 49.3 | 45.9 |
| Montana | 0.0 | 0.0 | 100.0 |
| Nebraska | 42.5 | 50.0 | 53.5 |
| Nevada | 45.9 | 58.8 | 53.4 |
| New Hampshire | 0.0 | 0.0 | 0.0 |
| New Jersey | 50.7 | 61.7 | 60.9 |
| New Mexico | 42.0 | 54.1 | 42.2 |
| New York | 51.6 | 71.7 | 69.2 |
| North Carolina | 37.4 | 46.6 | 48.9 |
| North Dakota | 0.0 | 0.0 | 0.0 |
| Ohio | 38.8 | 50.1 | 43.4 |
| Oklahoma | 42.1 | 51.4 | 48.0 |
| Oregon | 36.4 | 44.8 | 42.6 |
| Pennsylvania | 43.7 | 51.9 | 52.7 |
| Rhode Island | 37.1 | 57.7 | 50.8 |
| South Carolina | 39.2 | 50.7 | 63.0 |
| South Dakota | 0.0 | 0.0 | 0.0 |
| Tennessee | 41.0 | 49.4 | 69.0 |
| Texas | 39.6 | 51.0 | 43.6 |
| Utah | 0.0 | 0.0 | 33.3 |
| Vermont | 0.0 | 0.0 | 0.0 |
| Virginia | 45.8 | 54.4 | 60.5 |
| Washington | 43.2 | 49.0 | 44.0 |
| West Virginia | 45.5 | 79.6 | 0.0 |
| Wisconsin | 41.0 | 58.6 | 46.8 |
| Wyoming | 0.0 | 0.0 | 45.9 |
