# Supplementary material for: State-Level Variability in Location of Death of Patients with End-Stage Liver Disease
Source: Dig Dis Sci. 2025 Oct 8;71(3):933–40. doi: 10.1007/s10620-025-09433-w (PMC12982227; doi:10.1007/s10620-025-09433-w)
Supplement: Supplementary file 1 — Supplementary file1 (ZIP 1382 KB) [file 10620_2025_9433_MOESM1_ESM.zip › Supplementary/SDC Table 5.docx]

**Table 5**

*Proportion of Patients With End-Stage Liver Disease Who Died in a Hospice Facility*

| **State** | **Non- Hispanic/Latino White** | **Non- Hispanic/Latino Black or African American** | **Hispanic/Latino** |
| --- | --- | --- | --- |
| Alabama | 7.7 | 5.3 | 0.0 |
| Alaska | 0.0 | 0.0 | 0.0 |
| Arizona | 13.7 | 14.0 | 9.6 |
| Arkansas | 16.5 | 4.2 | 15.3 |
| California | 2.0 | 1.6 | 1.4 |
| Colorado | 11.5 | 12.7 | 10.9 |
| Connecticut | 4.4 | 0.0 | 6.4 |
| Delaware | 16.7 | 17.7 | 0.0 |
| District of Columbia | 0.0 | 5.5 | 0.0 |
| Florida | 28.9 | 17.9 | 20.6 |
| Georgia | 12.4 | 11.5 | 7.6 |
| Hawaii | 13.5 | 0.0 | 0.0 |
| Idaho | 6.7 | 0.0 | 0.0 |
| Illinois | 6.6 | 5.9 | 6.5 |
| Indiana | 6.8 | 3.1 | 8.7 |
| Iowa | 14.6 | 0.0 | 0.0 |
| Kansas | 12.6 | 18.3 | 13.0 |
| Kentucky | 11.3 | 7.6 | 0.0 |
| Louisiana | 9.4 | 9.7 | 11.6 |
| Maine | 15.4 | 0.0 | 0.0 |
| Maryland | 15.1 | 10.8 | 6.4 |
| Massachusetts | 6.5 | 0.0 | 0.0 |
| Michigan | 7.5 | 3.4 | 5.6 |
| Minnesota | 4.8 | 0.0 | 0.0 |
| Mississippi | 8.5 | 4.0 | 0.0 |
| Missouri | 4.7 | 4.9 | 12.2 |
| Montana | 6.9 | 0.0 | 0.0 |
| Nebraska | 3.9 | 0.0 | 0.0 |
| Nevada | 13.9 | 13.3 | 13.1 |
| New Hampshire | 25.7 | 0.0 | 0.0 |
| New Jersey | 25.3 | 4.5 | 2.5 |
| New Mexico | 30.8 | 0.0 | 5.2 |
| New York | 23.9 | 3.2 | 3.8 |
| North Carolina | 27.0 | 13.3 | 14.0 |
| North Dakota | 26.2 | 0.0 | 0.0 |
| Ohio | 23.0 | 8.4 | 15.4 |
| Oklahoma | 32.3 | 0.0 | 0.0 |
| Oregon | 38.6 | 0.0 | 0.0 |
| Pennsylvania | 26.5 | 6.6 | 5.8 |
| Rhode Island | 24.8 | 0.0 | 0.0 |
| South Carolina | 34.8 | 11.1 | 0.0 |
| South Dakota | 24.0 | 0.0 | 0.0 |
| Tennessee | 32.2 | 7.5 | 0.0 |
| Texas | 30.0 | 7.7 | 6.3 |
| Utah | 38.1 | 0.0 | 0.0 |
| Vermont | 23.5 | 0.0 | 0.0 |
| Virginia | 30.8 | 3.9 | 6.0 |
| Washington | 31.2 | 0.0 | 9.5 |
| West Virginia | 24.0 | 0.0 | 0.0 |
| Wisconsin | 26.3 | 8.6 | 14.7 |
| Wyoming | 27.6 | 0.0 | 0.0 |
