# Supplementary material for: State-Level Variability in Location of Death of Patients with End-Stage Liver Disease
Source: Dig Dis Sci. 2025 Oct 8;71(3):933–40. doi: 10.1007/s10620-025-09433-w (PMC12982227; doi:10.1007/s10620-025-09433-w)
Supplement: Supplementary file 1 — Supplementary file1 (ZIP 1382 KB) [file 10620_2025_9433_MOESM1_ESM.zip › Supplementary/SDC Table 6.docx]

**Table 6**

*Proportion of Location of Death of Patients With End-Stage Liver Who Died at Decedent's Home*

| **State** | **Non- Hispanic/Latino White** | **Non- Hispanic/Latino Black/African American** | **Hispanic/Latino** |
| --- | --- | --- | --- |
| Alabama | 35.5 | 23.5 | 50.8 |
| Alaska | 33.5 | 0.0 | 0.0 |
| Arizona | 31.8 | 27.1 | 29.9 |
| Arkansas | 28.2 | 23.4 | 34.7 |
| California | 31.8 | 21.9 | 27.7 |
| Colorado | 30.4 | 31.3 | 33.6 |
| Connecticut | 20.3 | 17.3 | 16.2 |
| Delaware | 28.2 | 21.9 | 0.0 |
| District of Columbia | 27.9 | 7.0 | 0.0 |
| Florida | 24.0 | 16.3 | 22.0 |
| Georgia | 32.7 | 21.1 | 24.5 |
| Hawaii | 26.9 | 0.0 | 50.0 |
| Idaho | 38.9 | 0.0 | 40.6 |
| Illinois | 27.8 | 17.1 | 23.0 |
| Indiana | 29.8 | 21.4 | 30.0 |
| Iowa | 26.0 | 37.1 | 23.6 |
| Kansas | 29.3 | 33.3 | 30.8 |
| Kentucky | 23.4 | 15.1 | 0.0 |
| Louisiana | 35.9 | 26.6 | 40.7 |
| Maine | 25.5 | 0.0 | 0.0 |
| Maryland | 23.7 | 14.8 | 24.0 |
| Massachusetts | 21.2 | 18.6 | 17.6 |
| Michigan | 30.1 | 19.0 | 28.7 |
| Minnesota | 30.6 | 29.2 | 21.4 |
| Mississippi | 34.7 | 26.2 | 43.5 |
| Missouri | 29.7 | 21.2 | 33.7 |
| Montana | 29.0 | 0.0 | 0.0 |
| Nebraska | 27.1 | 26.8 | 41.7 |
| Nevada | 26.0 | 23.0 | 20.2 |
| New Hampshire | 25.7 | 0.0 | 0.0 |
| New Jersey | 25.3 | 16.0 | 18.7 |
| New Mexico | 30.8 | 43.3 | 34.7 |
| New York | 23.9 | 10.5 | 14.9 |
| North Carolina | 27.0 | 20.9 | 28.0 |
| North Dakota | 26.2 | 0.0 | 0.0 |
| Ohio | 23.0 | 17.6 | 23.8 |
| Oklahoma | 32.3 | 29.7 | 32.9 |
| Oregon | 38.6 | 58.6 | 40.9 |
| Pennsylvania | 26.5 | 17.9 | 20.4 |
| Rhode Island | 24.8 | 0.0 | 0.0 |
| South Carolina | 34.8 | 24.4 | 30.5 |
| South Dakota | 24.0 | 0.0 | 0.0 |
| Tennessee | 32.2 | 20.8 | 24.5 |
| Texas | 30.0 | 20.9 | 31.9 |
| Utah | 38.1 | 0.0 | 42.5 |
| Vermont | 23.5 | 0.0 | 0.0 |
| Virginia | 30.8 | 21.6 | 27.1 |
| Washington | 31.2 | 28.2 | 29.2 |
| West Virginia | 24.0 | 0.0 | 0.0 |
| Wisconsin | 26.3 | 15.5 | 23.5 |
| Wyoming | 27.6 | 0.0 | 38.6 |
