# Supplementary material for: State-Level Variability in Location of Death of Patients with End-Stage Liver Disease
Source: Dig Dis Sci. 2025 Oct 8;71(3):933–40. doi: 10.1007/s10620-025-09433-w (PMC12982227; doi:10.1007/s10620-025-09433-w)
Supplement: Supplementary file 1 — Supplementary file1 (ZIP 1382 KB) [file 10620_2025_9433_MOESM1_ESM.zip › Supplementary/SDC Table 7.docx]

**Table 7**

*Proportion of Location of Death of Patients With End-Stage Liver Disease Who Died in a Hospice Facility or at Decedent's Home*

| **State** | **Non- Hispanic/Latino White** | **Non- Hispanic/Latino Black/African American** | **Hispanic/Latino** |
| --- | --- | --- | --- |
| Alabama | 43.2 | 28.8 | 50.8 |
| Alaska | 33.5 | 0.0 | 0.0 |
| Arizona | 45.6 | 41.1 | 39.4 |
| Arkansas | 44.7 | 27.6 | 50.0 |
| California | 33.8 | 23.6 | 29.1 |
| Colorado | 41.9 | 44.0 | 44.5 |
| Connecticut | 24.7 | 17.3 | 22.6 |
| Delaware | 44.9 | 39.6 | 0.0 |
| District of Columbia | 27.9 | 12.4 | 0.0 |
| Florida | 52.9 | 34.2 | 42.6 |
| Georgia | 45.1 | 32.6 | 32.1 |
| Hawaii | 40.4 | 0.0 | 50.0 |
| Idaho | 45.6 | 0.0 | 40.6 |
| Illinois | 34.3 | 23.0 | 29.4 |
| Indiana | 36.6 | 24.5 | 38.7 |
| Iowa | 40.6 | 37.1 | 23.6 |
| Kansas | 41.9 | 51.6 | 43.8 |
| Kentucky | 34.7 | 22.7 | 0.0 |
| Louisiana | 45.3 | 36.2 | 52.3 |
| Maine | 41.0 | 0.0 | 0.0 |
| Maryland | 38.8 | 25.6 | 30.4 |
| Massachusetts | 27.7 | 18.6 | 17.6 |
| Michigan | 37.6 | 22.4 | 34.3 |
| Minnesota | 35.4 | 29.2 | 21.4 |
| Mississippi | 43.1 | 30.2 | 43.5 |
| Missouri | 34.4 | 26.2 | 45.9 |
| Montana | 35.9 | 0.0 | 0.0 |
| Nebraska | 31.0 | 26.8 | 41.7 |
| Nevada | 39.9 | 36.3 | 33.3 |
| New Hampshire | 34.9 | 0.0 | 0.0 |
| New Jersey | 31.5 | 20.5 | 21.2 |
| New Mexico | 35.7 | 43.3 | 39.9 |
| New York | 31.7 | 13.8 | 18.7 |
| North Carolina | 45.6 | 34.3 | 42.0 |
| North Dakota | 26.2 | 0.0 | 0.0 |
| Ohio | 37.9 | 26.1 | 39.3 |
| Oklahoma | 37.6 | 29.7 | 32.9 |
| Oregon | 42.6 | 58.6 | 40.9 |
| Pennsylvania | 33.5 | 24.6 | 26.2 |
| Rhode Island | 42.5 | 0.0 | 0.0 |
| South Carolina | 48.2 | 35.5 | 30.5 |
| South Dakota | 40.0 | 0.0 | 0.0 |
| Tennessee | 37.5 | 28.3 | 24.5 |
| Texas | 39.2 | 28.6 | 38.3 |
| Utah | 38.1 | 0.0 | 42.5 |
| Vermont | 30.6 | 0.0 | 0.0 |
| Virginia | 34.9 | 25.5 | 33.0 |
| Washington | 39.4 | 28.2 | 38.7 |
| West Virginia | 35.6 | 0.0 | 0.0 |
| Wisconsin | 36.2 | 24.1 | 38.2 |
| Wyoming | 42.1 | 0.0 | 38.6 |
