# Supplementary material for: State-Level Variability in Location of Death of Patients with End-Stage Liver Disease
Source: Dig Dis Sci. 2025 Oct 8;71(3):933–40. doi: 10.1007/s10620-025-09433-w (PMC12982227; doi:10.1007/s10620-025-09433-w)
Supplement: Supplementary file 1 — Supplementary file1 (ZIP 1382 KB) [file 10620_2025_9433_MOESM1_ESM.zip › Supplementary/SDC Table 8.docx]

**Table 8**

*Proportion of Patients With End-Stage Liver Disease Who Died in a Medical Facility- Inpatient*

| **State** | **Non- Hispanic/Latino White** | **Non- Hispanic/Latino Black/African American** | **Hispanic/Latino** |
| --- | --- | --- | --- |
| Alabama | 46.8 | 61.0 | 49.2 |
| Alaska | 58.5 | 0.0 | 100.0 |
| Arizona | 37.5 | 58.9 | 46.8 |
| Arkansas | 41.2 | 60.2 | 50.0 |
| California | 48.6 | 60.0 | 57.2 |
| Colorado | 43.3 | 46.3 | 42.8 |
| Connecticut | 61.6 | 72.9 | 71.3 |
| Delaware | 43.1 | 60.4 | 100.0 |
| District of Columbia | 72.1 | 81.1 | 100.0 |
| Florida | 36.8 | 54.6 | 48.1 |
| Georgia | 42.0 | 56.8 | 61.6 |
| Hawaii | 35.0 | 65.0 | 50.0 |
| Idaho | 38.7 | 64.7 | 46.2 |
| Illinois | 47.3 | 0.0 | 58.1 |
| Indiana | 46.3 | 0.0 | 55.2 |
| Iowa | 41.9 | 62.9 | 76.4 |
| Kansas | 40.1 | 48.4 | 49.2 |
| Kentucky | 54.1 | 69.7 | 100.0 |
| Louisiana | 37.8 | 48.3 | 47.7 |
| Maine | 44.0 | 0.0 | 0.0 |
| Maryland | 47.2 | 62.7 | 69.6 |
| Massachusetts | 55.2 | 71.3 | 70.3 |
| Michigan | 48.4 | 65.4 | 53.1 |
| Minnesota | 42.8 | 59.9 | 64.3 |
| Mississippi | 43.3 | 58.4 | 56.5 |
| Missouri | 45.1 | 61.5 | 54.1 |
| Montana | 49.8 | 0.0 | 100.0 |
| Nebraska | 46.2 | 73.2 | 58.3 |
| Nevada | 49.7 | 63.7 | 58.0 |
| New Hampshire | 49.2 | 0.0 | 0.0 |
| New Jersey | 54.7 | 65.4 | 65.6 |
| New Mexico | 46.3 | 56.7 | 46.7 |
| New York | 56.0 | 78.4 | 73.2 |
| North Carolina | 42.3 | 53.4 | 58.0 |
| North Dakota | 57.4 | 0.0 | 0.0 |
| Ohio | 42.9 | 58.1 | 46.3 |
| Oklahoma | 46.6 | 55.2 | 60.6 |
| Oregon | 40.7 | 41.4 | 51.4 |
| Pennsylvania | 47.7 | 64.5 | 61.6 |
| Rhode Island | 41.9 | 100.0 | 100.0 |
| South Carolina | 42.4 | 54.7 | 69.5 |
| South Dakota | 41.3 | 0.0 | 0.0 |
| Tennessee | 44.9 | 59.6 | 75.5 |
| Texas | 43.5 | 58.7 | 48.4 |
| Utah | 38.9 | 0.0 | 40.4 |
| Vermont | 55.1 | 0.0 | 0.0 |
| Virginia | 50.2 | 61.0 | 67.0 |
| Washington | 48.5 | 71.8 | 54.9 |
| West Virginia | 49.2 | 100.0 | 0.0 |
| Wisconsin | 46.5 | 70.9 | 53.5 |
| Wyoming | 42.9 | 0.0 | 61.4 |
