# Supplementary material for: State-Level Variability in Location of Death of Patients with End-Stage Liver Disease
Source: Dig Dis Sci. 2025 Oct 8;71(3):933–40. doi: 10.1007/s10620-025-09433-w (PMC12982227; doi:10.1007/s10620-025-09433-w)
Supplement: Supplementary file 1 — Supplementary file1 (ZIP 1382 KB) [file 10620_2025_9433_MOESM1_ESM.zip › Supplementary/SDC Table 9.docx]

**Table 9**

*Proportion of Patients With Hepatocellular Carcinoma Who Died in a Hospice Facility*

| **State** | **Non- Hispanic/Latino White** | **Non- Hispanic/Latino Black/African American** | **Hispanic/Latino** |
| --- | --- | --- | --- |
| Alabama | 9.2 | 11.2 | 0.0 |
| Alaska | 0.0 | 0.0 | 0.0 |
| Arizona | 22.4 | 29.8 | 14.5 |
| Arkansas | 22.4 | 0.0 | 0.0 |
| California | 4.3 | 4.6 | 3.7 |
| Colorado | 19.1 | 22.0 | 21.8 |
| Connecticut | 10.2 | 0.0 | 0.0 |
| Delaware | 30.3 | 0.0 | 0.0 |
| District of Columbia | 0.0 | 14.6 | 0.0 |
| Florida | 35.7 | 27.1 | 27.2 |
| Georgia | 14.9 | 20.5 | 18.6 |
| Hawaii | 0.0 | 0.0 | 0.0 |
| Idaho | 9.1 | 0.0 | 0.0 |
| Illinois | 9.1 | 8.3 | 12.4 |
| Indiana | 9.9 | 9.1 | 0.0 |
| Iowa | 21.5 | 0.0 | 0.0 |
| Kansas | 16.7 | 0.0 | 0.0 |
| Kentucky | 19.0 | 19.4 | 0.0 |
| Louisiana | 11.9 | 13.3 | 0.0 |
| Maine | 28.0 | 0.0 | 0.0 |
| Maryland | 24.1 | 28.8 | 0.0 |
| Massachusetts | 12.4 | 0.0 | 13.3 |
| Michigan | 10.7 | 7.3 | 0.0 |
| Minnesota | 8.6 | 0.0 | 0.0 |
| Mississippi | 17.4 | 18.9 | 0.0 |
| Missouri | 8.6 | 13.7 | 0.0 |
| Montana | 6.0 | 0.0 | 0.0 |
| Nebraska | 0.0 | 0.0 | 0.0 |
| Nevada | 17.3 | 30.2 | 15.3 |
| New Hampshire | 17.7 | 0.0 | 0.0 |
| New Jersey | 10.1 | 7.5 | 9.4 |
| New Mexico | 9.0 | 0.0 | 12.4 |
| New York | 13.2 | 10.0 | 8.2 |
| North Carolina | 28.0 | 24.7 | 0.0 |
| North Dakota | 0.0 | 0.0 | 0.0 |
| Ohio | 22.4 | 17.6 | 28.8 |
| Oklahoma | 9.4 | 0.0 | 0.0 |
| Oregon | 4.2 | 0.0 | 0.0 |
| Pennsylvania | 13.9 | 17.7 | 13.3 |
| Rhode Island | 33.9 | 0.0 | 0.0 |
| South Carolina | 17.1 | 19.5 | 0.0 |
| South Dakota | 20.8 | 0.0 | 0.0 |
| Tennessee | 6.6 | 11.9 | 0.0 |
| Texas | 12.0 | 12.0 | 7.9 |
| Utah | 0.0 | 0.0 | 0.0 |
| Vermont | 18.3 | 0.0 | 0.0 |
| Virginia | 6.8 | 5.8 | 0.0 |
| Washington | 13.9 | 0.0 | 15.3 |
| West Virginia | 14.8 | 0.0 | 0.0 |
| Wisconsin | 17.7 | 28.3 | 0.0 |
| Wyoming | 14.0 | 0.0 | 0.0 |
